# Int6/eIF3e Silencing Promotes Placenta Angiogenesis in a Rat Model of Pre-eclampsia

**DOI:** 10.1038/s41598-018-27296-2

**Published:** 2018-06-12

**Authors:** Qin Li, Baolin Yao, Alexander Endler, Li Chen, Futoshi Shibasaki, Haidong Cheng

**Affiliations:** 1 Department of Gynecology, Obstetrics and Gynecology Hospital of Fudan University, No. 128, Shenyang Road, Shanghai, 200090 China; 2 Department of Obstetrics, Obstetrics and Gynecology Hospital of Fudan University, No. 128, Shenyang Road, Shanghai, 200090 China; 3 Department of Molecular Medical Research, Tokyo Metropolitan Institute of Medical Science, 2-1-6 Kamikitazawa, Setagaya-ku, Tokyo 156-8506 Japan; 4 Shanghai BIOMED Science Technology Co., Ltd, Room 2804-06 Baishu Building No. 1230, Zhongshan Bei Yi Road, Shanghai, 200437 China

## Abstract

We investigated whether stable eukaryotic translation initiation factor 3e/inter 6 (eIF-3e/Int6) RNA-silencing (siRNA-Int6) can ameliorate pre-eclampsia (PE) by promoting angiogenesis in an N-nitro-L-arginine methyl ester (L-NAME)-induced rat pre-eclampsia (PE) model. Twenty-four pregnant female Sprague–Dawley rats were allocated into 4 groups, including controls (Con) without any treatment, and 18 from gestational day (GD) 7 to GD17 L-NAME-treated rats, which were divided into stable siRNA-Int6 transfected (siRNA-Int6), negative vector control siRNA (NC-siRNA) and PE control (PE-Con) groups. All adenovirus siRNA transfections were performed on GD7 via intravenous tail injection. On GD0, GD11 and GD17, blood pressure, and on GD6 and GD17, protein estimations in 24 h urine samples were conducted. All animals were sacrificed on GD18. In the PE-Con group, placental Int6 was expressed to a significantly greater level than in the Con group, which was reversed by the application of siRNA-Int6. Blood pressure and proteinuria were significantly lower in the siRNA-Int6 group than in the PRE-Con group. As shown by CD31 and IB4 expression, placental micro-vascular density (MVD) was significantly higher in the siRNA-Int6 group than in the PE-Con and NC-siRNA groups, which has accompanied by enhanced trophoblast invasion. Int6 silencing alleviated the maternal clinical manifestations of pre-eclampsia and promoted placental angiogenesis in pregnant L-NAME-treated rats.

## Introduction

Pre-eclampsia (PE) is a pregnancy-specific disorder characterized by maternal endothelial dysfunction and inhibition of angiogenesis. It eventually causes placental ischemia and is clinically characterized by hypertension and proteinuria, appearing for the first time after 20 weeks of gestation and affects approximately 2 to 8% of pregnant women worldwide^1–4^. PE is a crucial cause of maternal and infant morbidity and mortality^5,6^. The underlying etiology and pathogenesis of this disorder is not well understood, but it has been noted that in women with pre-existing vascular disease, PE occurs more frequently and maternal vascular dysfunction is intensified by placental factors^7^. Most research supports the theory that PE is characterized by an imbalance of angiogenic vascular endothelial growth factor (VEGF), placental growth factor (PlGF), transforming growth factor-β (TGF-β) and anti-angiogenic (soluble fms-like tyrosine kinase-1 (sFlt-1) and soluble endoglin (sEng)) factor activity, leading to placental vascular impairment and under-perfusion^8–10^. Overexpression of sFlt-1, which is an antagonist of VEGF and PlGF, has been suggested to inhibit angiogenesis, particularly during early placental development^11,12^. An altered sFlt-1/PlGF expression ratio has been proposed to serve as a PE marker^13–15^. However, in a recent publication, Llurba *et al*. (2015) suggested that PE should be regarded as a three-stage process in which the first stage is defined as deficient expression of pro-angiogenic factors such as VEGF, PlGF and hypoxia-inducible factors (HIFs), which leads to abnormal remodeling of spiral arteries and trophoblast invasion in the decidua^16^. In previous research, it has been demonstrated that the eukaryotic translation initiation factor 3e/inter 6 (eIF-3e/Int6) downregulates the endothelial PAS domain-containing protein 1/hypoxia-inducible factor 2α (EPAS1/HIF2α) and its silencing led to potent neo-angiogenesis in a diabetic mouse wound healing model via stabilization of HIF2α protein under normoxia^17,18^. In the present study, we investigated whether HIF2α stabilization via Int6-silencing might have an influence on PE in a rat model.

## Materials and Methods

### Animal studies and therapeutic regimes

Approval was obtained from the Animal Ethics Committee of the Fudan University and all procedures involving animals were performed in accordance with the guidelines for the humane treatment of laboratory animals (Ministry of Science and Technology of the People’s Republic of China, Policy No. 2006 398).

Five to seven week-old, female Sprague–Dawley rats, weighing 210–250 g were provided by Chinese Slaccas Inc. (Shanghai, China). Each animal was put into an individual metabolic cage (Kangwei Inc. Shanghai, China) with water *ad libitum*.

They copulated with weight-matched male rats at a female:male ratio of 2:1 during periods of sexual excitement and activity. We took vaginal smears on the second morning and then carried out Papanicolaou staining, and observed the specimens using an optical microscope. We confirmed the 0th gestational day by estrum and the presence of sperm. The protocol was approved by the ethics boards of Obstetrics and Gynecology Hospital of Fudan University.

**Figure 1:**
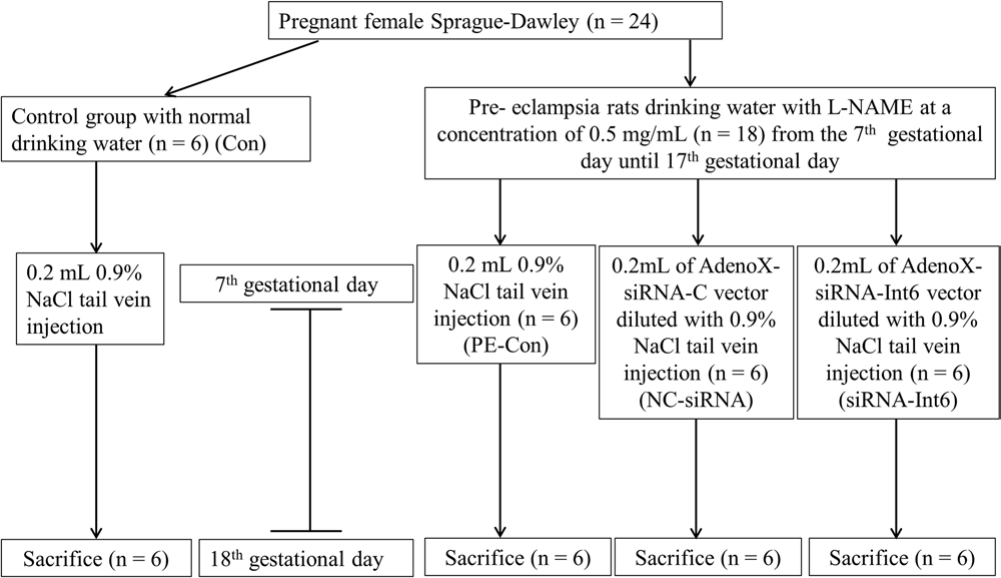
Flowchart of the present study.

Twenty-four pregnant female Sprague–Dawley rats were divided into 4 groups. The control group (Con) (n = 6) received filtered drinking water and 0.2 mL 0.9% NaCl injected into the tail vein on the 7th GD. The PE group received drinking water with L-NAME (Sigma, St. Louis, MO, USA) at a concentration of 0.5 mg/mL from the 7th GD for 10 days and were divided into a PE control (PE-Con) group (n = 6), which received 0.2 mL 0.9% NaCl by tail vein injection on the 7th GD, an AdenoX-siRNA-Int6 vector treated group (siRNA-Int6, n = 6) and an AdenoX-siRNA-negative control vector treated group (NC-siRNA, n = 6), in which the recombinant adenoviruses were injected into the tail vein (5 × 10^8^ plaque forming units) on the 7th GD (Fig. 1).

### Vector constructs for stable siRNA-Int6 transfection

The siRNA-Int6 (pRNAT-U6) construct was prepared according to the manufacturer’s instructions (GeneScript Corp, China) with the insert sequence 5′-CAATTACAGAATTTGTTGA-3′ (human (nn 972–990, NCBI Reference Sequence: XM_017013398.1) and rat (nn 921–939, NCBI Reference Sequence: XM_017603441.1)), which has been evaluated for efficacy before by transient transfection of HeLa cells; the random-sequence siRNA (negative control) was purchased from the manufacturer (Ambion, Waltham, MA, UK). Both constructs were integrated into the Adeno-X Adenoviral System 3 and recombinant adenoviruses were created with a Clonetech In-fusion kit (Clontech Laboratories, Mountain View, CA, USA) according to the manufacturer’s instructions.

### Blood pressure measurement and urine analysis

The systolic blood pressure (SBP) of each animal was determined on the 0th, 11th and 17th GDs using the non-invasive tail-cuff method and a BP-2000 Blood Pressure Analysis System (Visitech Systems, Inc., Apex, NC, USA). Every rat was pre-measured for training for 3 consecutive days prior to the 0th GD. Each rat was preheated for 5 min to 38 °C before each measurement was taken, 3 times for averaging. The 24-hour urine output of each animal was collected on the 6^th^ and 17^th^ GDs. Proteinuria was detected using CBB kits purchased from the Jiancheng Institute of Biotechnology (Nanjing, China), according to the manufacturer’s instructions.

### Immunohistochemistry analysis

**Figure 2:**
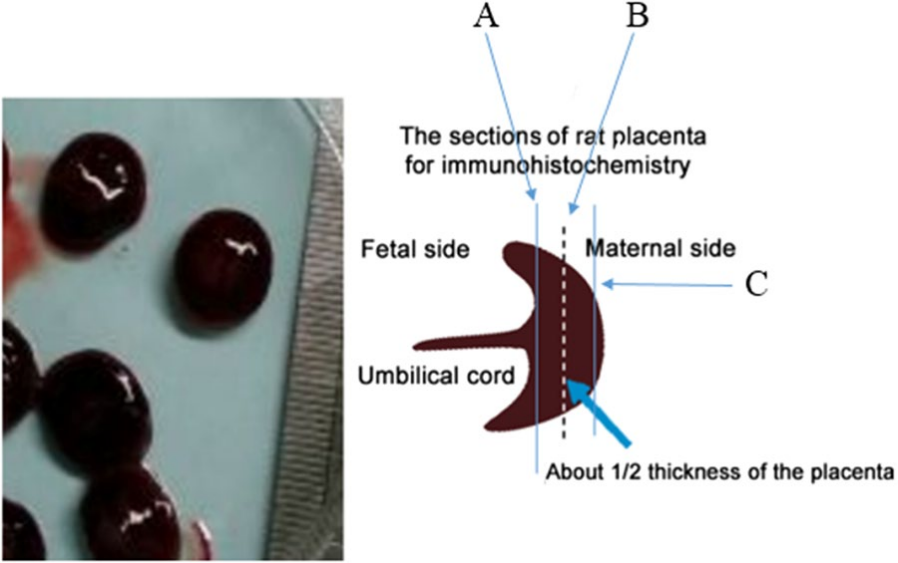
Scheme of the placental tissue sampling for immunohistochemistry (IHC) analyses. Section A is located close to the fetal, section B in the center and section C close to the maternal side of the placenta.

The placentas were collected and fixed in paraformaldehyde. The paraffin-embedded placental samples were sliced into 3-μm thick sections, which were divided into 3 categories namely: A) close to the fetal side, B) in the center, and C) close to the maternal side of the placentas (Fig. 2).

The placental tissue sections were handled for immunohistochemistry (IHC) as previously described^19^. Briefly, after deparaffinization, hydration, antigen repairing, decreased endogenous peroxidase activity and blockade with 3% normal bovine serum, the sections were incubated with either CD31 antibodies (Abcam, ab119339, 1:200, Cambridge, MA, USA), Int6 antibodies (Abcam, ab36766, 1:1000, Boston, MA, USA), IB4 antibodies (sc-65254, Santa Cruz Biotechnology, USA.) and basic cytokeratin antibodies (3G129, sc-70402, Santa Cruz Biotechnology, USA.) at 4 °C overnight.

The secondary antibodies (1:100) anti-mouse IgG (Cat. No YS1004) and anti-rabbit IgG (H + L) (Cat. No YS1001) were purchased from Detai Biologics Co., Ltd. (Nanjing, China). The HRP/DAB detection IHC kits were purchased from BestBio Biological Technology Co. Ltd. (Shanghai, China). The staining area was viewed with a BX53 + DP70 Olympus microscope (Olympus (China) Co., Ltd., Beijing, China) and photographed with a digital camera.

As in previously described studies, Int6 immunoreactivity grading was based on the German Immunoreactive-score^20^. First, staining intensity in the cytoplasm was rated on a scale from 0 to 3, with 0 being no staining, 1 weak staining, 2 moderate staining and 3 strong staining. Then, positive and negative cells were counted. The percentage of positive cells was then scored as: no staining as 0; 1–10% as 1; 11–50% as 2; 51–80% as 3 and 81–100% as 4. The final score was calculated by multiplying the score obtained with the staining intensity by that derived from the percentage of positive cells, achieving results ranging from 0 to 12. A final score of 0 was regarded as negative, 1–4 as weak, 5–8 as moderate and 9–12 was considered as strong immunoreactivity.

### Enzyme-linked immunosorbent assay (ELISA) method

After general anesthesia, 1 mL of venous blood was collected from the rats, and the samples centrifuged for 10 min at 3,000 r/min. The separated serum was stored at −70 °C. Int-6, HIF2α, interleukin (IL)-6, IL-8 and basic fibroblast growth factor (bFGF) were measured using ELISA (eBioscience, San Diego, CA, USA) according to the manufacturer’s instructions.

### Microvessel density

Microvessel density (number of vessels) was determined by image analysis of CD31-immunostained sections. The identification of microvessels did not require them to have a complete lumen and erythrocytes. It could be regarded as an autocephalous vessel as long as there were dyed, brown, vascular endothelial cells or vascular endothelial cell clusters separated from the adjacent vessels, trophocytes and mesenchyme. Any vessels with thick muscular walls that could contain erythrocytes were not counted.

One investigator performed microvessel density (MVD) assessments according to the Weidner’s method^21^, with 3 high-power fields (×200 magnification) of the placenta analyzed and then averaged.

### Statistical analysis

Statistical analysis was carried out using SPSS statistics for Windows (version 17.0, Chicago, SPSS Inc). Differences in the relative expression levels of each indicator in the 4 groups were analyzed using a Mann-Whitney test. SBP changes in each group at different times were analyzed using an ANOVA test, and *P* < 0.05 was considered to be statistically significant.

## Results

**Figure 3:**
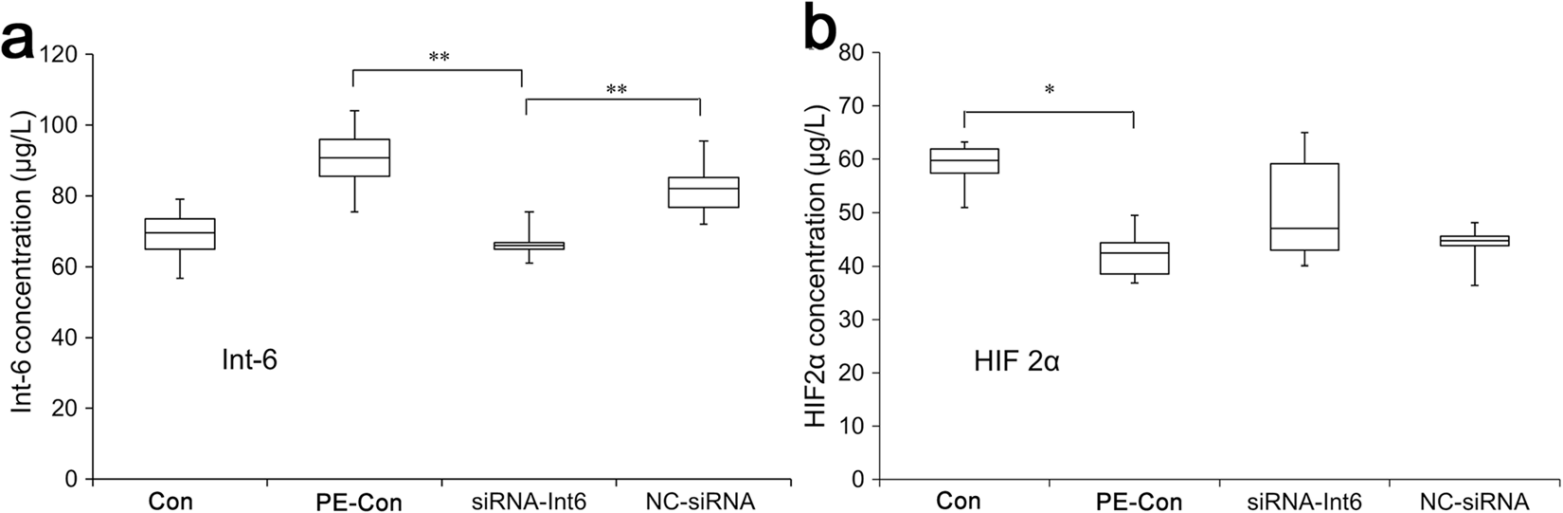
Blood serum concentrations of Int6 and HIF2α. **P < 0.01, *P < 0.05. Con = control without pre-eclampsia; PE-Con = pre-eclampsia control without treatment; siRNA-Int6 = pre-eclampsia rats treated with siRNA against Int6; NC-siRNA = pre-eclampsia rats treated with siRNA vector negative control.

**Figure 4:**
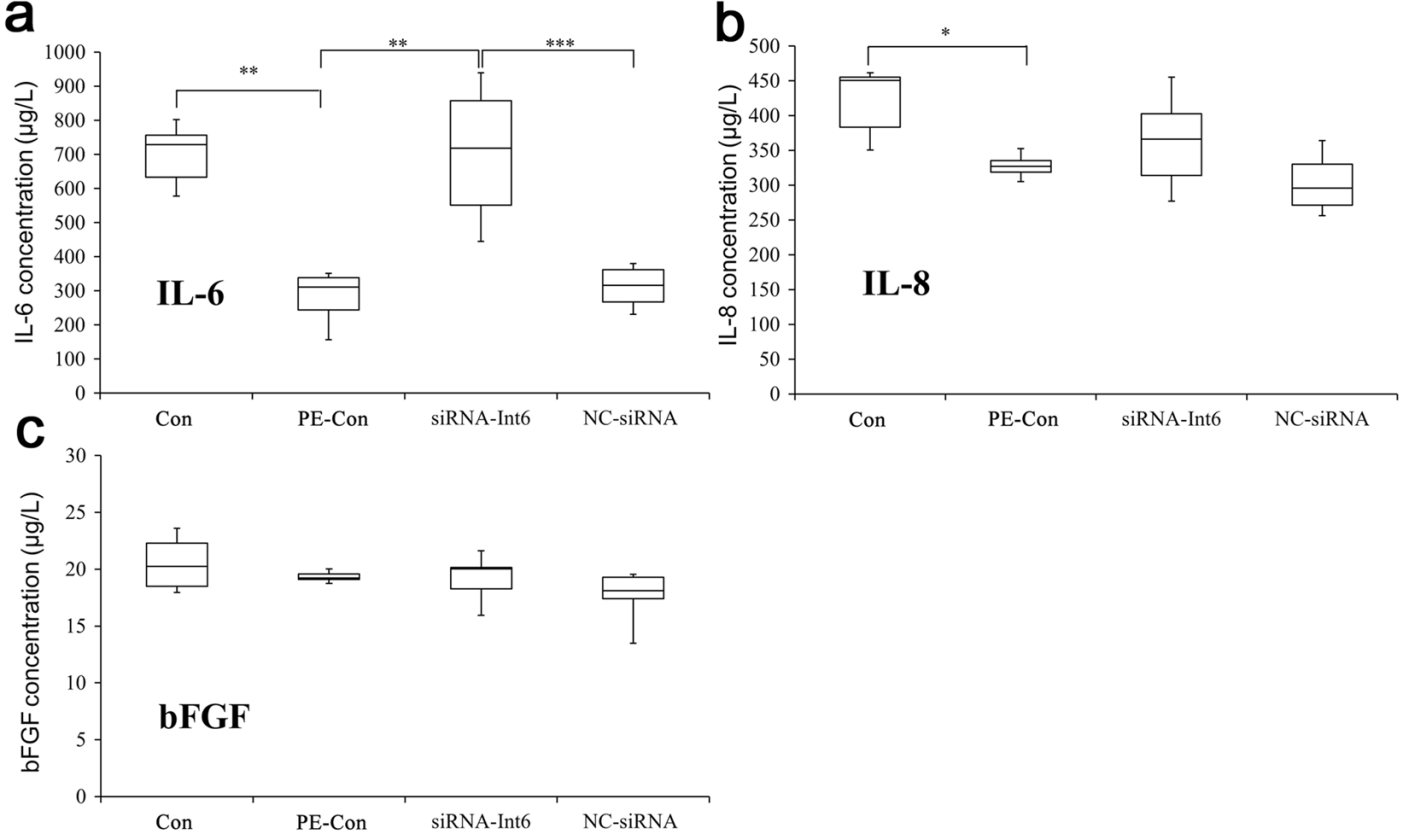
Blood serum concentrations of HIF2α related cytokines IL-6 and IL-8 and bFGF. ***P < 0.001, **P < 0.01, *P < 0.05. Con = control without pre-eclampsia; PE-Con = pre-eclampsia control without treatment; siRNA-Int6 = pre-eclampsia rats treated with siRNA against Int6; NC-siRNA = pre-eclampsia rats treated with siRNA vector negative control.

We measured the serum protein concentrations of HIF2α and Int-6 (Fig. 3) as well as IL-6, IL-8 and bFGF (Fig. 4) in the 4 groups. In the PE-Con and NC-siRNA rats Int-6 was significantly upregulated, was and the upregulation was significantly inhibited by siRNA Int-6 (Fig. 3a), a trend which was also visible for HIF2α. However, in the siRNA-Int6 group, the apparent enhancement of serum HIF2α concentrations did not reach statistical significance (Fig. 3b). In the serum of PE-Con rats the concentrations of IL-6 and IL-8 were significantly decreased compared to the Con group (Fig. 4a,b), which was significantly restored by siRNA-int6 for IL-6, whereas Int-6 silencing had no effect on bFGF serum concentrations (Fig. 4c).

### Influences on PE clinical manifestations after siRNA-Int6 treatment

**Figure 5:**
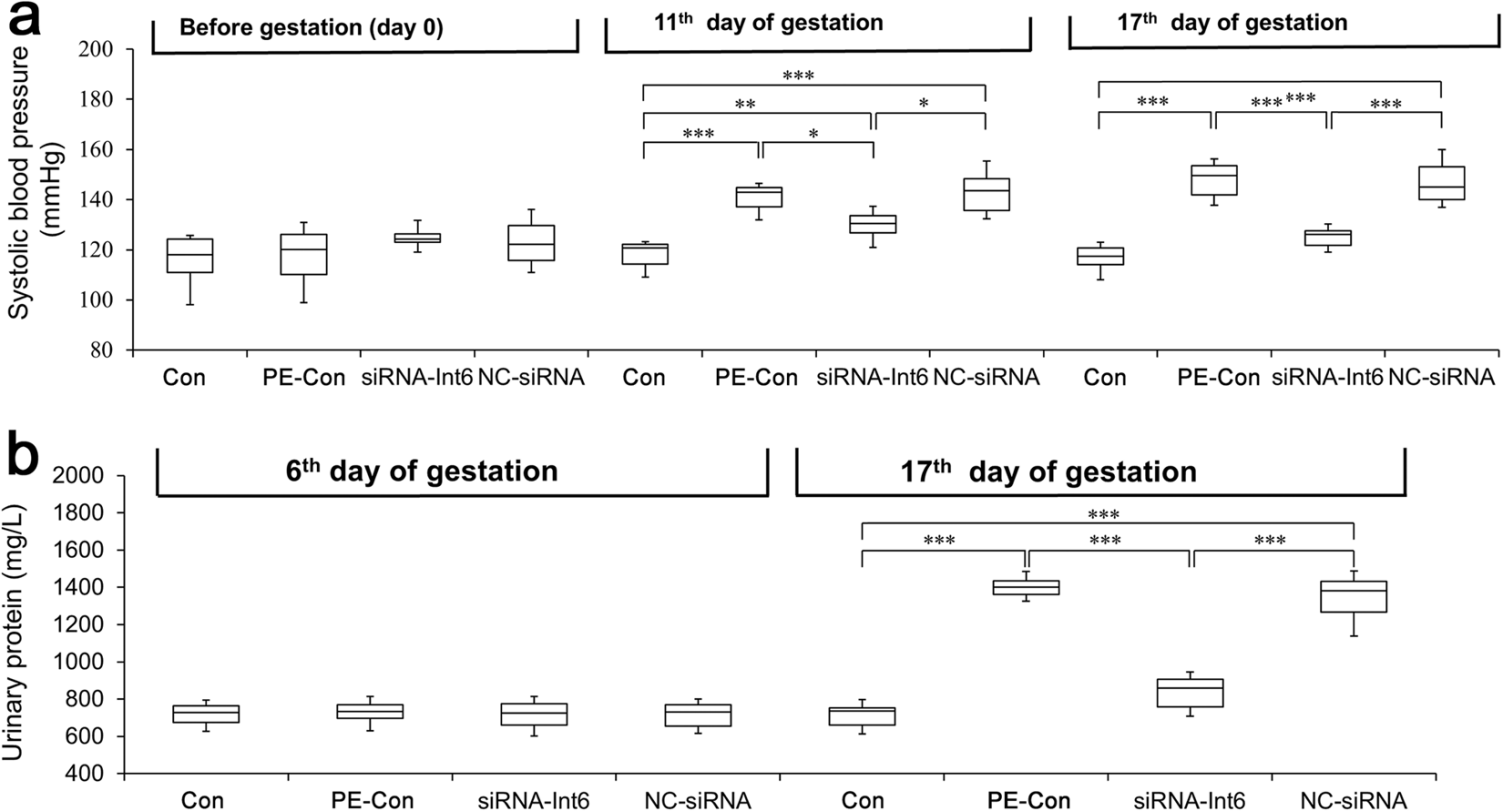
(**a**) SBP of each indicated group was measured non-invasively using the tail-cuff method on GD0, GD11 and GD17. (**b**) The proteinuria in each group was detected using CBB kits on GD6 and GD17. ***P < 0.001, **P < 0.01, *P < 0.05. Con = control without pre-eclampsia; PE-Con = pre-eclampsia control without treatment; siRNA-Int6 = pre-eclampsia rats treated with siRNA against Int6; NC-siRNA = pre-eclampsia rats treated with siRNA vector negative control.

We measured the SBP in GD0, GD11 and GD17 rats, respectively (Fig. 5a). The baseline of SBP values was not significant different among each group on GD0 (*P* > 0.05). On GD11, compared with the Con group (118.13 ± 5.93 mmHg) the SBP was significantly increased in the PE-Con group (140.78 ± 5.79 mmHg) (*P* < 0.001) and the vector control group (141.27 ± 9.11 mmHg) (*P* < 0.001) as well as in the siRNA-Int6 group (129.88 ± 5.89 mmHg), but to a smaller extent (*P* < 0.05). On GD17, the SBP was again significantly higher in the PE-Con (147.85 ± 7.61 mmHg) and the NC-siRNA (146.83 ± 9.14 mmHg) groups compared with the Con group (116.75 ± 5.55 mmHg) (*P* < 0.001). However, the SBP was not significantly different between the Con (116.75 ± 5.55 mmHg) and the siRNA-Int6 groups (125.33 ± 7.03 mmHg) (*P* > 0.05) at that time (Fig. 5a). In accordance with the SBP, the degree of proteinuria was not significantly different (*P* > 0.05) between each group on GD6. Significant differences were noted among all groups on GD17 with the Con group being (740.03 ± 53.29 mg/L) vs the PE-Con group (1,451.4 ± 242.96 mg/L) (*P* < 0.001); siRNA-Int6 group (775.71 ± 89.45 mg/L) vs PE-Con group (1,451.4 ± 242.96 mg/L) (*P* < 0.001) and the Con group (740.03 ± 53.29 mg/L) vs siRNA-Int6 group (775.71 ± 89.45 mg/L) (*P* > 0.05) (Fig. 5b). The results showed that the SBP was significantly elevated by the 4^th^ day after rats drank water containing L-NAME, which indicated that the PE animal model was successfully established. The clinical manifestations of PE were improved after siRNA-Int6 treatment and the effect of siRNA-Int6 on SBP was more obvious with an increase in the gestational age.

### Expression of Int6 in placentas

**Figure 6:**
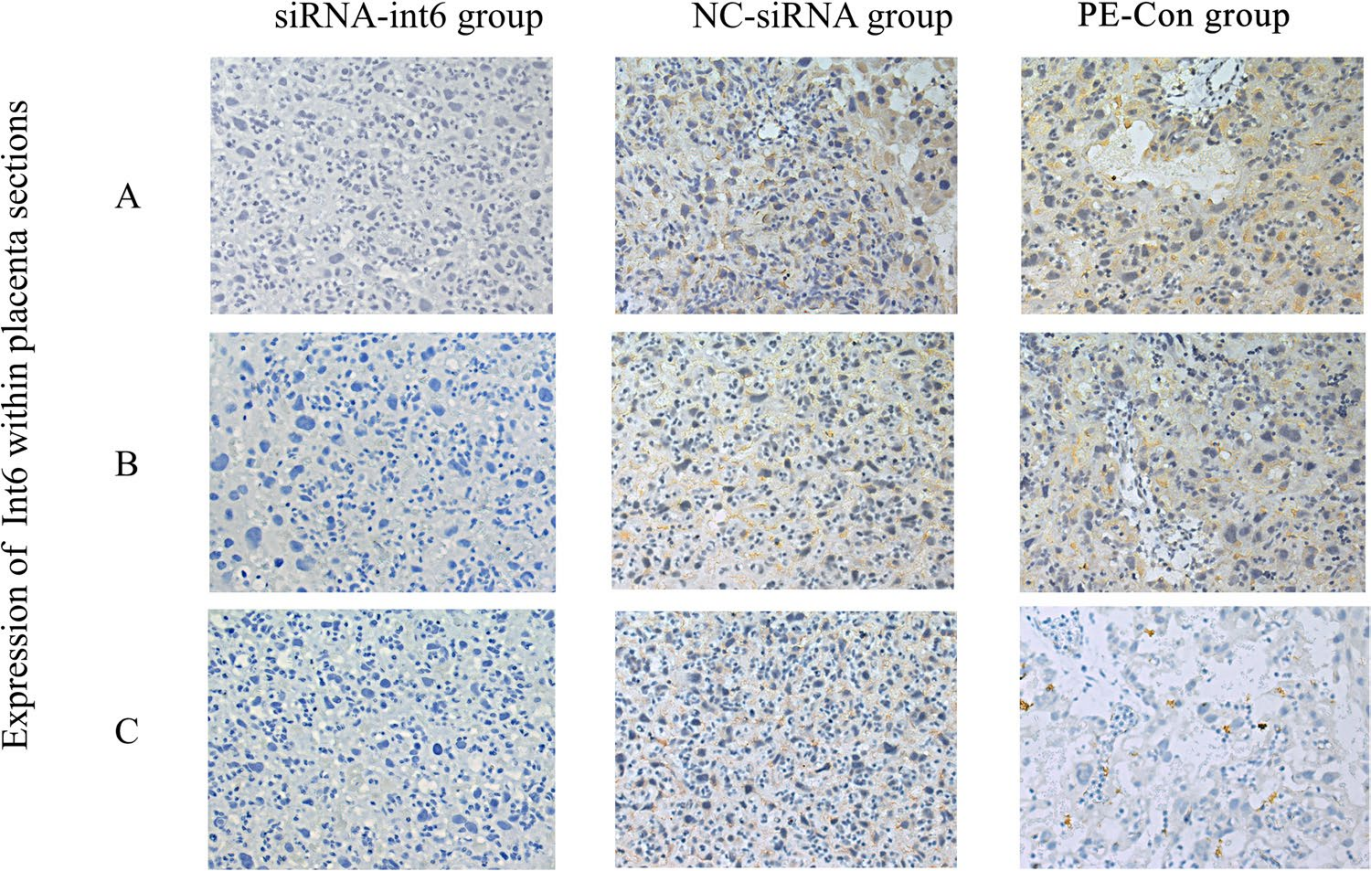
Int6 expression in placental rat tissues derived from (**A**) close to the fetal side, (**B**) in the center and (**C**) close to the maternal side of the placentas. PE-Con = pre-eclampsia control without treatment; siRNA-Int6 = pre-eclampsia rats treated with siRNA against Int6; NC-siRNA = pre-eclampsia rats treated with siRNA vector negative control.

We took samples from 3 different placental areas and examined the distribution of Int-6 expression. As shown in Fig. 6, Int6 was least expressed in the siRNA-Int6 group and expressed to the greatest degree in the siRNA-NC and PE groups, particularly in the A and B sections, which were isolated from the maternal and central areas of the placenta.

**Figure 7:**
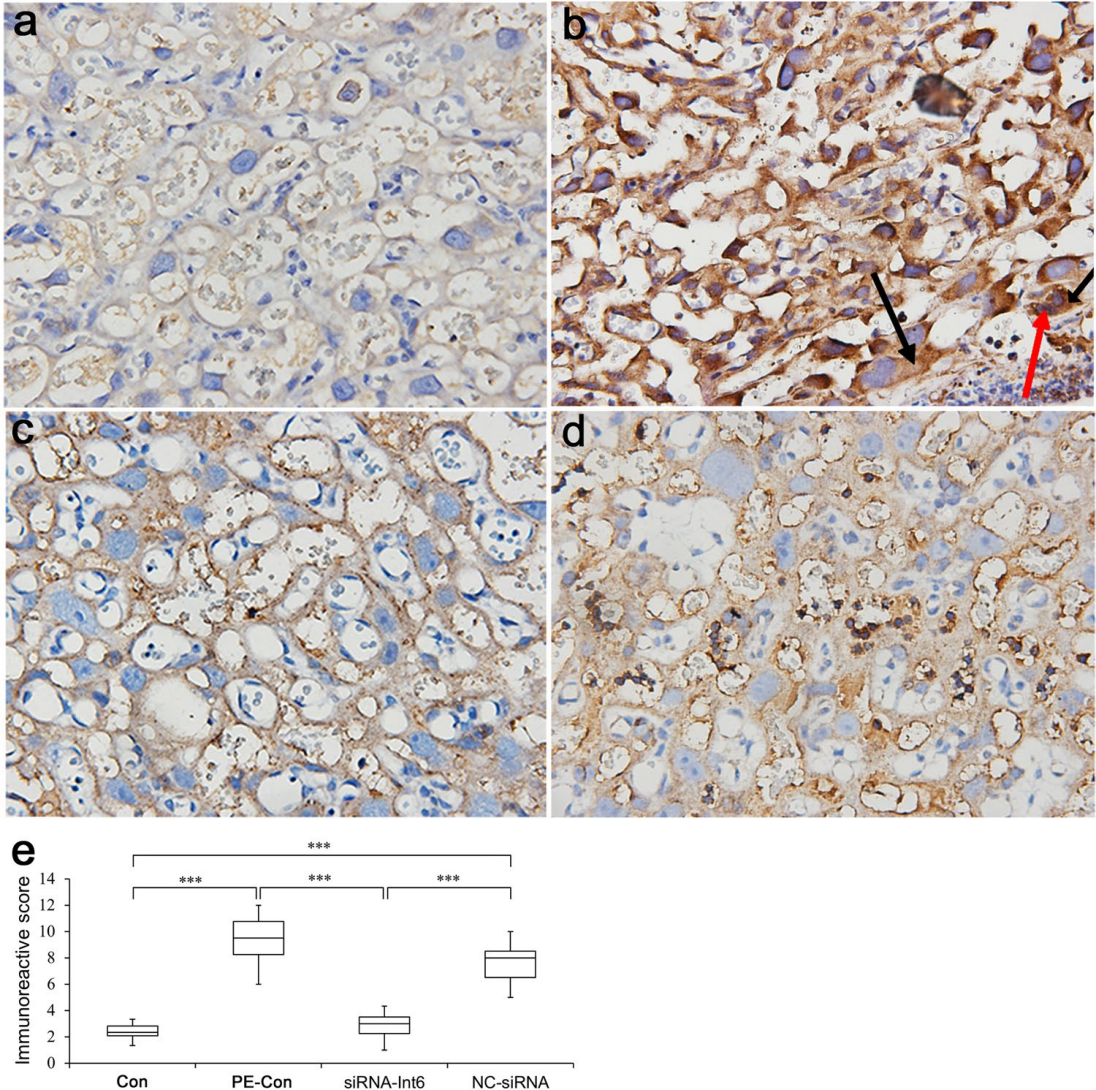
Immunohistochemical staining of Int-6 in placental tissues (section B) (blue is DAPI dye of the nuclei and brown is the Int-6 dye). (**a**–**d**) Represent the Con, PE-Con, siRNA-Int6 and NC-siRNA groups. Black arrows in (**b**) indicate cytosolic Int-6 and the red arrow indicate nucleic Int-6. (**e**) Immunoreactivity scores of the indicated groups. *P < 0.001. Con = control without pre-eclampsia; PE-Con = pre-eclampsia control without treatment; siRNA-Int6 = pre-eclampsia rats treated with siRNA against Int6; NC-siRNA = pre-eclampsia rats treated with siRNA vector negative control.

We took samples from the boundary between the maternal and fetal sides (section B) of the placenta and examined Int-6 expression in nuclei and cytosol of the trophoblasts using immunohistochemistry. As shown in Fig. 7b, PE led to enhanced Int6 expression and nucleic accumulation, which was reversed by Int-6 silencing and the immunoreactivity scores showed significantly reduced Int-6 staining in the siRNA-Int6 group (Fig. 7e).

### The effect of siRNA-Int6 on placentas

**Figure 8:**
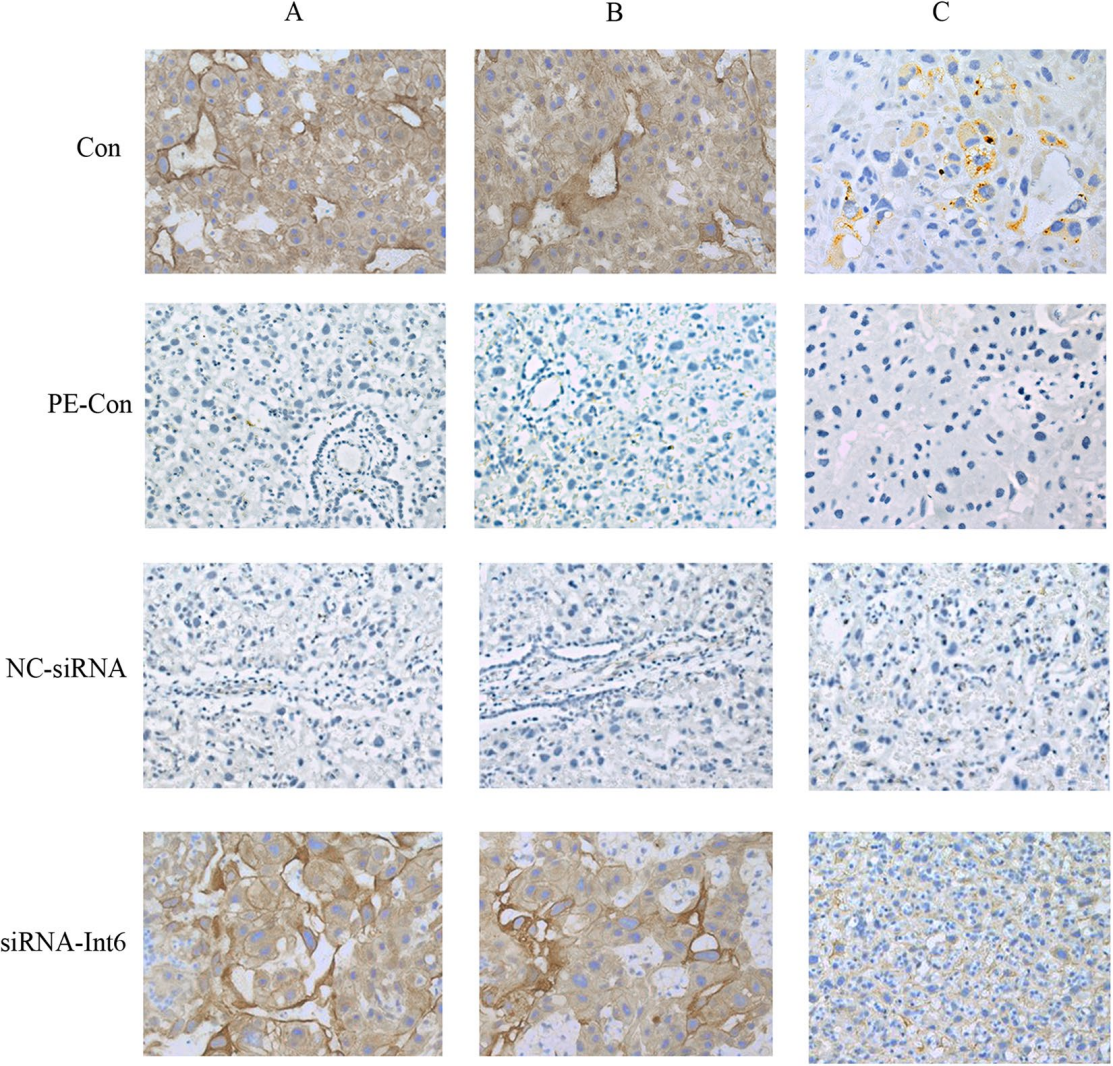
CD31 immunostaining in Con, PE-Con, NC-siRNA and siRNA-Int6 in slices derived from (**A**) close to the maternal side, (**B**) in the center and (**C**) close to the fetal side of rat placentas. Con = control without pre-eclampsia; PE-Con = pre-eclampsia control without treatment; siRNA-Int6 = pre-eclampsia rats treated with siRNA against Int6; NC-siRNA = pre-eclampsia rats treated with siRNA vector negative control.

Figure 8 shows that the expression of CD31, a marker for endothelial cells, was highest in the Con as well as in the siRNA-Int6 A and B sections of the placenta. Minor expression was found in the region close to the fetal side of the placenta (section C) in the siRNA-Int6 and the Con groups.

**Figure 9:**
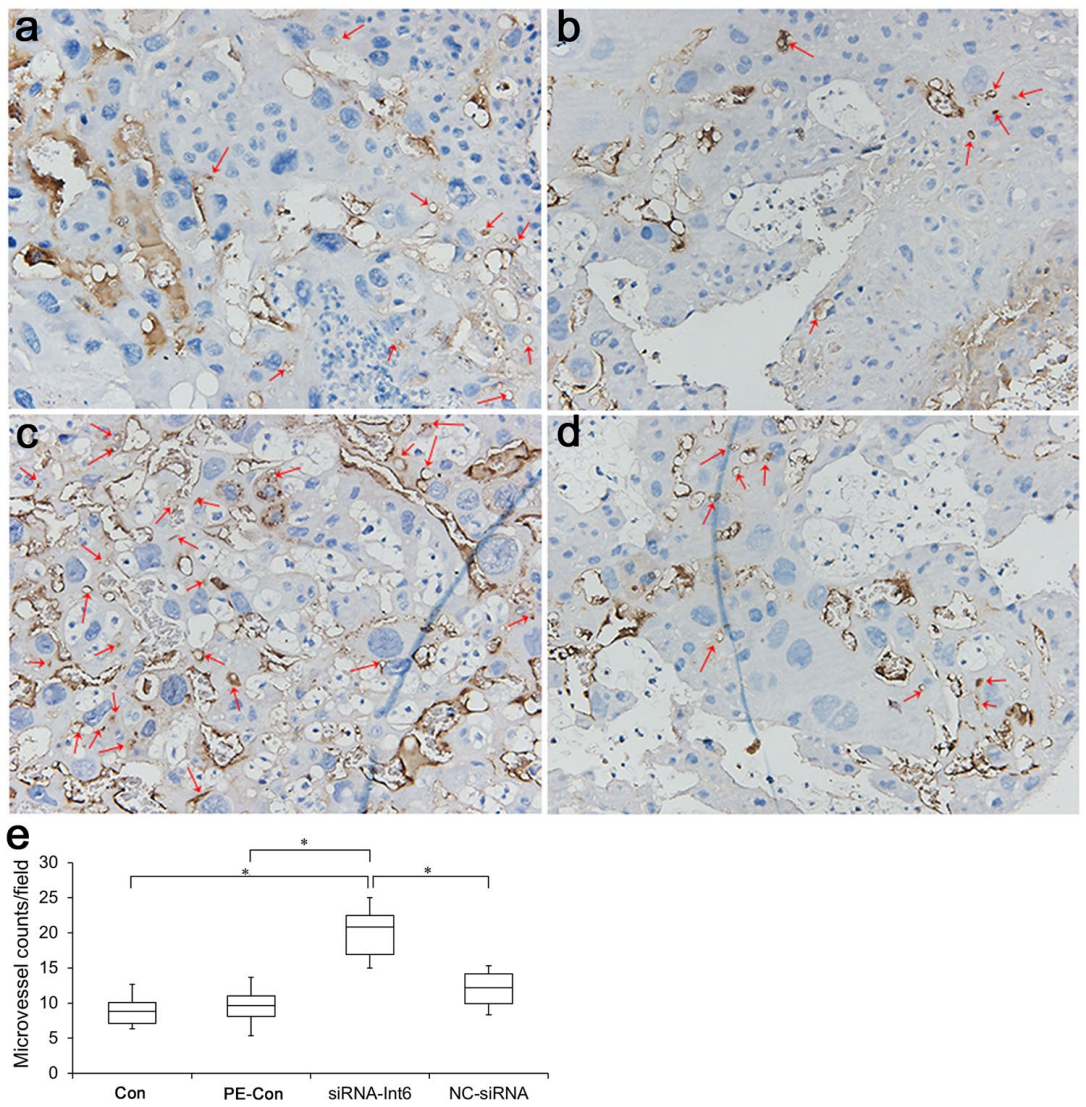
CD31 immunostaining of placental tissue to reveal microvessels. A greater degree of small vessel neoformation (arrows) is visible in the siRNA-Int6 group compared to the other groups. (**a**–**d)** Represent the Con, PE-Con, siRNA-Int6 and NC-siRNA groups. E. Statistical analysis revealed that there was a clear trend toward increased MVD in the siRNA-Int6 group. *P < 0.05. Con = control without pre-eclampsia; PE-Con = pre-eclampsia control without treatment; siRNA-Int6 = pre-eclampsia rats treated with siRNA against Int6; NC-siRNA = pre-eclampsia rats treated with siRNA vector negative control.

To investigate an effect of siRNA-Int6 on placental vascularization, CD31 stained microvessels in B sections were counted in high power fields (200×). The results showed that the MVD was significantly increased in the siRNA-int6 group compared to the PE-Con and Con groups (Fig. 9) (P < 0.05).

**Figure 10:**
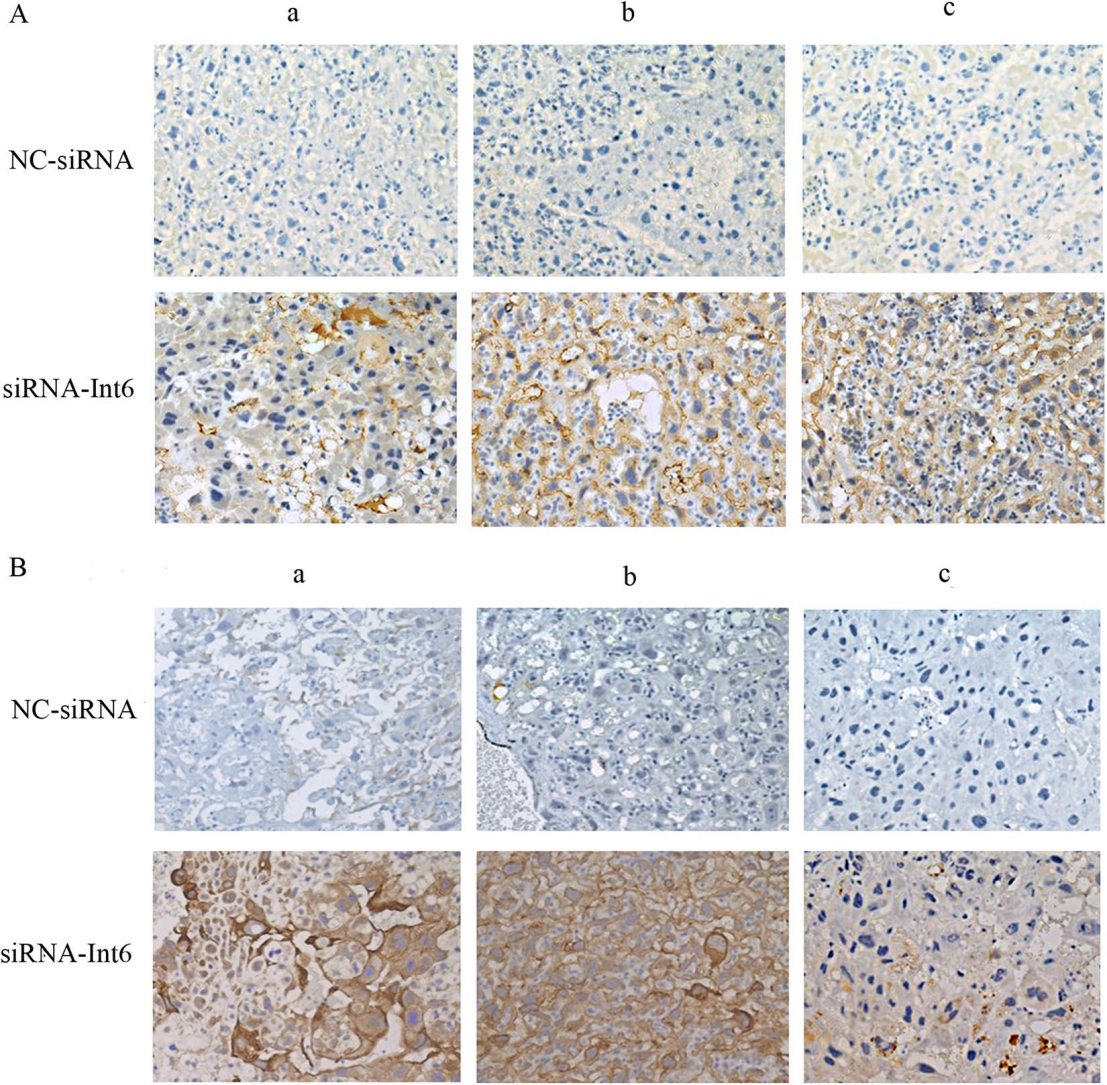
IB4 (**A**,**B**) cytokeratin immunostaining in close to maternal (a) center (b) and close to the fetal sides (c) of placentas from si-RNA-Int6 and NC-siRNA treated rats. siRNA-Int6 = pre-eclampsia rats treated with siRNA against Int6; NC-siRNA = pre-eclampsia rats treated with siRNA vector negative control.

Similar to CD31, also staining intensities of IB4, a vascular endothelial cell marker and cytokeratin, a trophoblast marker were greater in all placental sections of the siRNA-Int6 treated group compared to the NC-siRNA group (Fig. 10).

### Influence on pregnancy outcomes after siRNA-Int6 treatment

**Figure 11:**
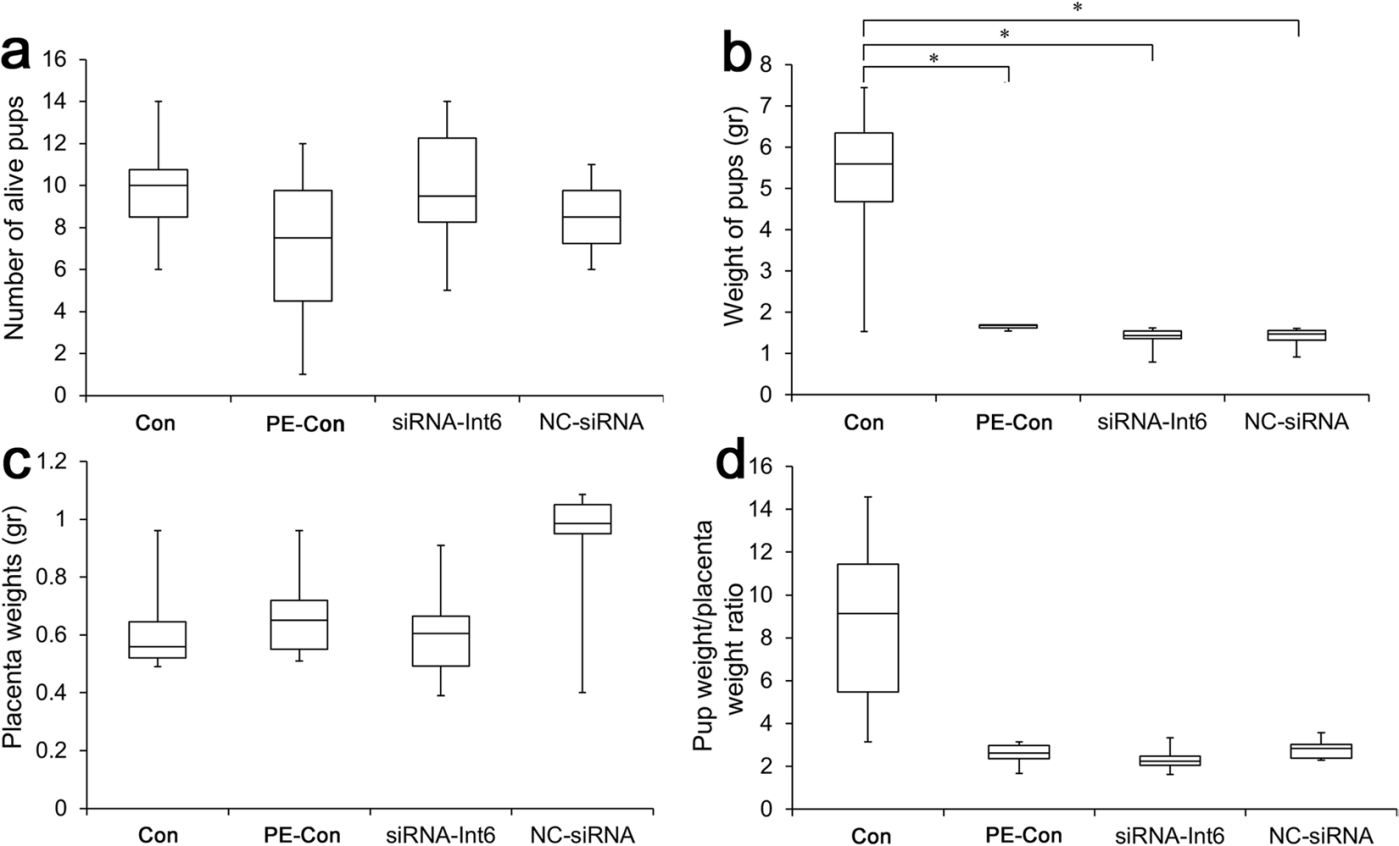
Influence on pregnancy outcomes. (**a**) The number of alive pups, (**b**) The weight of the pups (gr), (**c**) The placenta weights (gr), (**d**) Ratio of placenta and pup weights. *P < 0.05. Con = control without pre-eclampsia; PE-Con = pre-eclampsia control without treatment; siRNA-Int6 = pre-eclampsia rats treated with siRNA against Int6; NC-siRNA = pre-eclampsia rats treated with siRNA vector negative control.

We chose various indexes to determine pregnancy outcomes, mainly the weight of the pups, the number of living pups, as well as placental weight. All data were collected directly after cesarean section. There were no significant differences between each group regarding the number of living pups (Fig. 11a), but the weight of the pups were significantly higher only in the Con group compared to the other groups (*P* < 0.05) (Fig. 11b). The average weight of the placentas was not significantly different between the groups (Fig. 11c), a finding also reflected in the pup weight/placenta weight ratio (Fig. 11d), with a virtually identical pattern to pup weight.

## Discussion

Previous studies have demonstrated that PE is associated with shallow extra villous trophoblast (EVT) invasion into the decidua and poor vascular remodeling, which leads to reduced uteroplacental blood flow^22,23^. Ultimately, the placenta becomes ischemic and then an insufficient blood supply leads to continued hypoxic conditions when sFlt-1 and sEng are upregulated^24,25^, and stabilized HIF1α^26^ suppresses cytotrophoblast invasion via increased tumor growth factor-β3 (TGF-b3) expression^27–29^. Since sEng, a soluble receptor of TGF-β1, impairs binding to its receptors, the vasorelaxation activity of TGF-β1 is suppressed and in PE a vicious circle of placental hypoxia is maintained^8^.

An advantage of our study was that HIF2α activity could be selectively enhanced without changing HIF1α activity, which is unavoidable with hypoxia. Int-6 was identified as a negative feedback regulatory factor of HIF2α, which specifically binds to and degrades HIF2α via the ubiquitin/proteasome pathway even under hypoxic conditions^18^. Various studies have demonstrated that Int-6 silencing could promote angiogenesis and enhance wound healing by upregulating the expression of HIF2α^17,30–32^. Chen *et al*. (2010) reported that Int6 silencing caused a stronger effect on angiogenesis than overexpression of HIF2α^17^.

In our study, PE led to significantly reduced levels of maternal HIF2α, IL-6 and IL-8, the later factor being both transcribed by HIF2α^30^, as well as significantly enhancing Int-6 serum concentrations, indicating, that at least in a L-NAME-induced rat PE model HIF2α signaling is changed systemically, while siRNA-Int6 reversed these changes partly and led to inhibition of hypertension and proteinuria in PE rats (Fig. 5). Similar reductions of blood pressure and urinary protein levels were obtained in a previous study, in which the constitutively developing PE rat BPH/5 was stably transfected with an adenovirus coding VEGF_121_^33^. In another previous study, selective overexpression of HIF2α in pre-eclampsic placentas was reported^34^. Since HIF2α activity is regulated by negative feedback regulation of its stability caused by HIF2α-induced transcription of Int6 via hypoxia-response elements^17^, the upregulation of Int6 in placentas of the PE-Con rats could be explained, but it is unclear why physiologically upregulated HIF2α did not lead to angiogenesis in pre-eclampsic placentas as observed by Rajakumar *et al*.^34^.

Other research has suggested, that the key point for hypoxic dysfunction of trophoblasts is the overexpression of HIF2α in pre-eclampsic placentas^35^. However, based on the present results, we do not agree that enhanced HIF2α protein levels in placentas are a PE-causing factor, since Int6 silencing could enhance MVD in the PE rats as shown by the upregulated vascularization marker CD31 and IB4 (Figs 8, 9 and 10A), which was accompanied by trophoblast invasion as shown by enhanced expression of the trophoblast marker cytokeratin^36^ (Fig. 10B).

Rather, we suggest that restricted causative or resulting HIF2α activity is the cause of insufficient angiogenesis in the PE placenta, which is supported by the fact that HIF2α has been recognized as an important factor for trophoblast differentiation and functions^37^.

Triggering of neovascularization did not change pup weight in our experiments, which might have been due to PE-independent effects of L-NAME on the rat fetuses, since L-NAME can cross the placental barrier^38^.

The limitations of our study was that (1) the transfection of siRNA-Int6 was not restricted to the rat placentas (2) that systemic HIF2α activity might have influenced the outcome and (3) the small sample size. In addition, in the present pilot study, commonly investigated PE-induced pathological changes in the placenta, such as the placental depth ratio, quantification of placental total vessel length, vascular density and branching index within the placenta, were not determined.

## Conclusion

Stable Int6 silencing in a PE rat model led to enhanced placental vascularization and avoidance of maternal hypertension and urinary protein development.
